# Total-body imaging of mu-opioid receptors with [^11^C]carfentanil in non-human primates

**DOI:** 10.1007/s00259-024-06746-2

**Published:** 2024-05-09

**Authors:** Chia-Ju Hsieh, Catherine Hou, Hsiaoju Lee, Cosette Tomita, Alexander Schmitz, Konstantinos Plakas, Jacob G. Dubroff, Robert H. Mach

**Affiliations:** Department of Radiology, Perelman School of Medicine, University of Pennsylvania, Philadelphia, Pennsylvania 19104 USA

**Keywords:** Mu-opioid receptors, [^11^C]carfentanil, Naloxone, GSK1521498, Positron emission tomography, PennPET explorer

## Abstract

**Purpose:**

Mu-opioid receptors (MORs) are widely expressed in the central nervous system (CNS), peripheral organs, and immune system. This study measured the whole body distribution of MORs in rhesus macaques using the MOR selective radioligand [^11^C]carfentanil ([^11^C]CFN) on the PennPET Explorer. Both baseline and blocking studies were conducted using either naloxone or GSK1521498 to measure the effect of the antagonists on MOR binding in both CNS and peripheral organs.

**Methods:**

The PennPET Explorer was used for MOR total-body PET imaging in four rhesus macaques using [^11^C]CFN under baseline, naloxone pretreatment, and naloxone or GSK1521498 displacement conditions. Logan distribution volume ratio (DVR) was calculated by using a reference model to quantitate brain regions, and the standard uptake value ratios (SUVRs) were calculated for peripheral organs. The percent receptor occupancy (%RO) was calculated to establish the blocking effect of 0.14 mg/kg naloxone or GSK1521498.

**Results:**

The %RO in MOR-abundant brain regions was 75–90% for naloxone and 72–84% for GSK1521498 in blocking studies. A higher than 90% of %RO were observed in cervical spinal cord for both naloxone and GSK1521498. It took approximately 4–6 min for naloxone or GSK1521498 to distribute to CNS and displace [^11^C]CFN from the MOR. A smaller effect was observed in heart wall in the naloxone and GSK1521498 blocking studies.

**Conclusion:**

[^11^C]CFN total-body PET scans could be a useful approach for studying mechanism of action of MOR drugs used in the treatment of acute and chronic opioid use disorder and their effect on the biodistribution of synthetic opioids such as CFN. GSK1521498 could be a potential naloxone alternative to reverse opioid overdose.

**Supplementary Information:**

The online version contains supplementary material available at 10.1007/s00259-024-06746-2.

## Introduction

With the dramatic rise in the availability and abuse of illicit opioids, the mortality rate of overdose associated with opioid use disorder has reached epidemic proportions in the world [1, 2], particularly the United States [3, 4]. Most of the synthetic opioids are mu-opioid receptor (MOR) agonists with the potential of causing respiratory depression [5]. Naloxone is a highly effective opioid antagonist that has been widely used to reverse the effect of opioids [6, 7]. GSK1521498 is a MOR selective antagonist or conditional inverse agonist [8, 9] that has been studied in alcohol use disorder and binge eating in obesity [8, 10, 11]; it has also been suggested as an alternative to naloxone in the rescue of opioid overdose [12]. Current studies of GSK1521498 in the treatment of binge-eating disorders and compulsive alcohol seeking have used either oral or intraperitoneal administration of the drug [8–10, 13, 14] to evaluate the long-lasting pharmacokinetics for MORs. The acute effects of GSK1521498 by intramuscular (IM), intravenous (IV), or intranasal administration to determine the ability to reverse the effects of opioids for MORs has not yet been studied.

MORs are widely expressed in the central nervous system (CNS), peripheral organs, and immune system [15, 16]. The mechanism of MORs in brain have been extensively studied in vivo by using positron emission tomography (PET) imaging with radioligands such as [^11^C]carfentanil ([^11^C]CFN) in human [17–22], non-human primates (NHPs) [23, 24], and rodents [25]. However, only a small number of in vivo studies regarding the function and the density of MORs in the spinal cord and peripheral organs have been reported [26].

In this study, we demonstrated the distribution of total-body MORs in both CNS and peripheral organs of rhesus macaques by using the PennPET Explorer, long axial field of view instrument, with the MOR radioligand [^11^C]CFN under baseline and opioids blockade conditions. The effects of opioid blockade by naloxone via IM administration prior to the [^11^C]CFN injection, and naloxone or GSK1521498 given by IV administration during the [^11^C]CFN scans, were conducted to evaluate the effect of the MOR antagonists on the pharmacokinetics of [^11^C]CFN in the CNS, spinal cord, and peripheral organs. The comparison of receptor occupancy (RO) and the rate of target engagement for GSK1521498 and naloxone were also evaluated.

## Materials and methods

### Non-human primates and study design

A series of sequential baseline, retest, and naloxone pretreatment (0.14 mg/kg, IM administration 10 min prior to [^11^C]CFN injection) [^11^C]CFN imaging studies were performed on the same four male rhesus macaques (16–24 years old, body weight 6.0–17.7 kg). Naloxone displacement (0.14 mg/kg, IV administration at 40 min post [^11^C]CFN injection) scans were performed on one of the 4 non-human primates (NHPs), and displacement scans of GSK1521498 (0.14 mg/kg, IV administration at 40 min post [^11^C]CFN injection) were performed on 3 of the 4 NHPs. Details of the studies on each NHP are listed on Supplementary Table 1. NHPs were fasted for 12 h prior to each [^11^C]CFN study, and initially anesthetized by IM injection with ketamine (4 mg/kg) and dexmedetomidine (0.05 mg/kg). Each NHP was intubated and anesthesia maintained with 0.75-2% isoflurane in 1–2 L/min oxygen. A percutaneous catheter was placed for the [^11^C]CFN, naloxone, or GSK1521498 injection. Body temperature was maintained with a recirculating water warming pad and vital signs such as blood pressure, pulse oximetry, and EKG were monitored continuously during the [^11^C]CFN scan. All animal experiments in this study were performed under protocols approved by the University of Pennsylvania Institutional Animal Care and Use Committee (IACUC).

### Preparation of [^11^C]CFN

[^11^C]CO_2_ was produced by the IBA cyclotron at the University of Pennsylvania by a ^14^N (p,α) ^11^C reaction on an 0.5% O_2_ in N_2_ gas target. Briefly, [^11^C]CO_2_ was trapped in a liquid nitrogen cooled tube in the Synthra MeIPlus synthesis module (Synthra GmbH, Germany). [^11^C]CO_2_ was converted to [^11^C]CH_4_ using a molecular sieve, nickel and hydrogen. [^11^C]CH_4_ was converted to [^11^C]CH_3_I using iodine in a heated loop and bubbled into the reaction mixture containing desmethylcarfentanil, dissolved in DMF. The solution was heated to 60 °C for 5 min. The resulting radiolabeled products are purified with a C2 Bond Elut cartridge (Agilent, US) after dilution with 1% ammonium hydroxide solution. The product was eluted with ethanol and followed by wash with sterile saline. The final product formulation is filtered through a 0.22 μm filter before collecting into a sterile final product vial. The finished products were tested for chemical and radiochemical purity by HPLC analysis (Supplementary QC test of [^11^C]CFN). The specific activity was 480.5 ± 401.2 MBq/nmol.

### Preparation of GSK1521498

GSK1521498 was prepared according to the patent literature [27]. It was characterized by proton NMR, carbon NMR, and HRMS (Supplementary Characterization of GSK1521498). The molecule was formulated as a hydrochloric acid salt prior to use in the in vivo studies.

### *[*^*11*^*C]CFN PET data acquisition*

Each [^11^C]CFN PET scan was conducted on the PennPET Explorer [28, 29], a PET scanner with a 142 cm axial field of view that capable of doing total-body dynamic acquisition studies. A low dose CT scan was performed to confirm positioning and attenuation correction followed by 90 or 120 min dynamic PET image acquired in list-mode after venous injection of 105.3 ± 8.5 MBq of [^11^C]CFN (injected mass: 102.8 ± 207.2 ng/kg). For the baseline, retest, and naloxone pretreatment scans, 90 min dynamic images were acquired in 29 time frames (12 × 10 s, 2 × 30 s, 4 × 60 s, 2 × 120 s, 3 × 180 s, 2 × 300 s, 2 × 600 s and 2 × 1200 s). For the displacement studies, naloxone or GSK1521498 was given at 40 min post [^11^C]CFN injection, and the dynamic scan was continued for a total of 120 min in 48 time frames (12 × 10 s, 2 × 30 s, 4 × 60 s, 2 × 120 s, 3 × 180 s, 4 × 300 s, 7 × 120 s, 4 × 240 s, and 10 × 300 s). All PET images were reconstructed using time-of-flight list-mode ordered subsets expectation maximization (OSEM, 25 subsets) reconstruction algorithm [28]. The reconstructed images had a matrix size of 300 × 300 × 712 and a voxel size of 2 × 2 × 2 mm^3^.

### Image analysis

All total-body PET/CT images were processed and analyzed by using Pmod software (version 4.2, PMOD Technologies Ltd., Zurich, Switzerland). Each perfusion phase (1–10 min) of brain PET image was co-registered to the corresponding MR-T1 weighted brain image. The individual MR-T1 weighted brain image was spatially normalized to the D99 rhesus macaque MR brain template [30]. Then, the spatial normalization parameters were applied to the corresponding dynamic brain PET image to form a spatially normalized brain PET image in the D99 template domain. Fifteen volumes of interest (VOIs) modified from the D99 macaque brain atlas including thalamus, caudate, putamen, nucleus accumbens, midbrain, medulla, hippocampus, amygdala, prefrontal, anterior cingulate, posterior cingulate, temporal, parietal, visual cortex, and cerebellar cortex were used for further PET neuroimaging analyses. Additional VOIs of spinal cord and peripheral organs including cervical, thoracic, and lumbar spinal cord, spinal bone marrow, heart wall, upper arm skeletal muscle, liver, spleen, bilateral kidneys, and small intestine were manually delineated on the PET or CT image of each scan.

Time-activity curves (TACs) of each VOI were extracted from dynamic PET images by calculating standardized uptake values (SUVs). A total-body VOI of each scan was used to determine the injected activity to calibrate the residual dose in the percutaneous catheter post [^11^C]CFN injection, since the percutaneous catheter could not be removed to measure the residual dose during the dynamic PET scan. Distribution volume ratio (DVR) for each brain VOI and cervical spinal cord was computed from Logan graphical analysis [19] by using cerebellar cortex as the reference tissue with an average tissue-to-plasma clearance rate, k2’, derived from a simplified reference tissue model. The DVR for baseline, retest, and naloxone pretreatment studies were calculated by using 0–90 min TAC. For the displacement studies with naloxone or GSK1521498, [^11^C]CFN was injected and a dynamic acquisition initiated. This acquisition consisted of two phases, 0–40 min (before MOR antagonist administration) and 40–120 min (after MOR antagonist administration). TACs of each VOI were used to calculate the respective DVR for control and blockade to determine the effect of the blocking agent. The SUV ratios (SUVRs) of all the CNS VOIs to the cerebellar cortex at each time window were also calculated.

The percent variation (%Var) between baseline and retest in DVR for brain and cervical spinal cord, and 70–90 min SUVR (SUVR_70 − 90 min_) for spinal cord and peripheral organs were calculated to evaluate the test-retest variability.

$$\%\text{V}\text{a}\text{r}=100\times \frac{\text{Baseline }{\text{DVR}}-\text{Retest }{\text{DVR}}}{(\text{Baseline }{\text{DVR}}+\text{Retest }{\text{DVR}})/2}$$

or

$$\%\text{V}\text{a}\text{r}=100\times \frac{\text{Baseline }{\text{SUVR}}_{70-90\text{min}}-\text{Retest }{\text{SUVR}}_{70-90\text{min}}}{(\text{Baseline }{\text{SUVR}}_{70-90\text{min}}+\text{Retest }{\text{SUVR}}_{70-90\text{min}})/2}$$

The percent receptor occupancy (%RO) was calculated to determine the blocking effect of naloxone pretreatment study by the following equation:

$$\%\text{RO}=100\times [1-\frac{{\text{DVR}}_{\text{Pretreatment}}-1}{{\text{DVR}}_{\text{Baseline}}-1}]$$

The %RO of blocking effect for naloxone or GSK1521498 displacement study was calculated by the following equation:

$$\%\text{RO}=100\times [1-\frac{{\text{DVR}}_{40-120\text{min}}-1}{{\text{DVR}}_{0-40\text{min}}-1}]$$

The percent difference of SUVR_70 − 90 min_ (%Diff_SUVR_) between the baseline/retest and naloxone pretreatment studies was calculated to determine the naloxone blocking effect for spinal cord and peripheral organs.

$$\%{\text{Diff}}_{\text{SUVR}}=100\times \left[1-\frac{\text{Pretreatment }{\text{SUVR}}_{70-90\text{min}}}{\text{Baseline }{\text{SUVR}}_{70-90\text{min}}}\right]$$

The percent reduction (%Reduction) of SUV between 40 and 70 min was also calculated to compare the displacement effect between baseline/retest and the displacement studies.

$$\%\text{Re}\text{duction}=100\times \left[1-\frac{{\text{SUV}}_{70\text{min}}}{{\text{SUV}}_{40\text{min}}}\right]$$

### Statistical analysis

All statistical analyses were performed on GraphPad Prism software, version 7.02 (GraphPad Inc., San Diego, CA). A paired t-test was used to evaluate the test-retest variability and blocking effects by comparing the differences of DVRs and SUVR_70 − 90 min_. A two-tailed P value < 0.05 was considered statistically significant.

## Results

### Determination of the reference region for non-human primate brain imaging studies

**Fig. 1:**
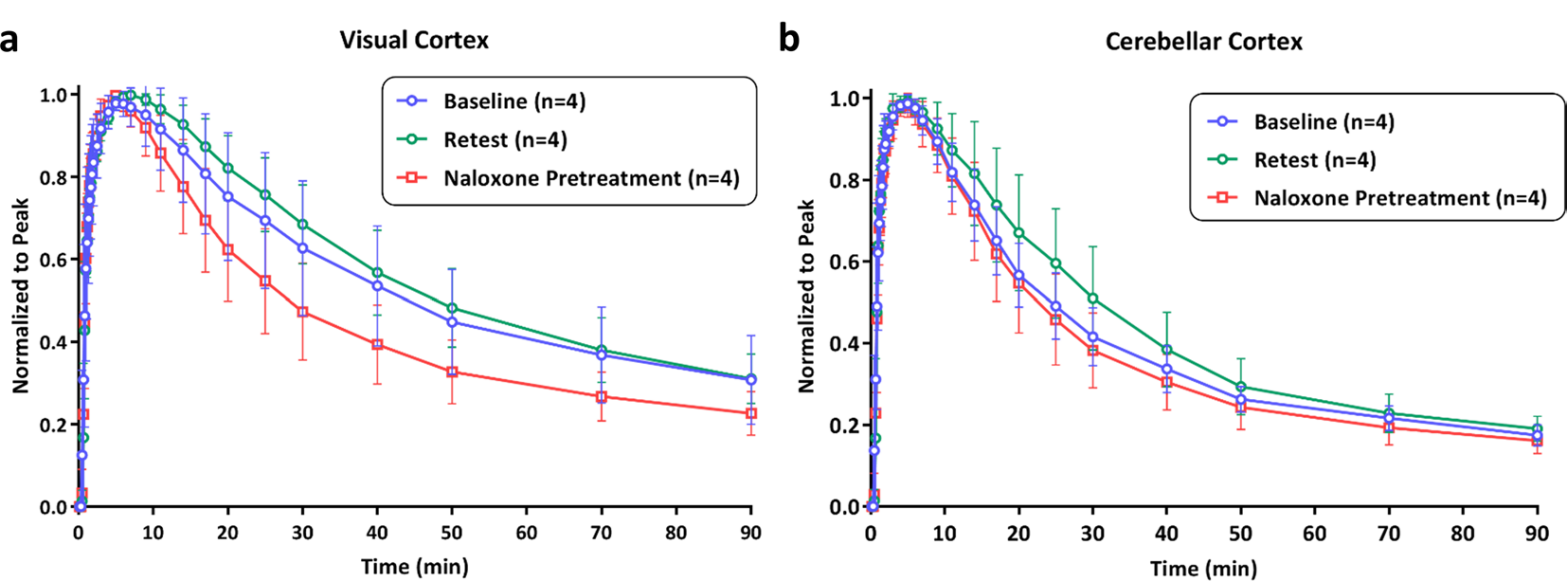
TACs of normalized to peak uptake for (**a**) visual cortex and (**b**) cerebellar cortex in baseline, retest, and naloxone pretreatment studies. Data points present as mean ± standard deviation

The TACs, normalized to peak radiotracer uptake, are shown in Fig. 1 for two regions in rhesus macaque brain with a low binding of [^11^C]CFN, the visual cortex (Fig. 1a) and cerebellar cortex (Fig. 1b). A faster clearance rate of [^11^C]CFN in the visual cortex was observed in the naloxone pretreatment study (Fig. 1a) relative to the baseline and retest studies. The average clearance (i.e., half-time) rates of [^11^C]CFN in visual cortex were 44.8 ± 18.0 min for the test study, 48.8 ± 12.5 min for the retest study, and 29.1 ± 9.2 min for naloxone pretreatment study. For the cerebellar cortex (Fig. 1b), the clearance rates of [^11^C]CFN were similar for baseline, retest, and naloxone pretreatment studies. The average clearance half-time of [^11^C]CFN for baseline, retest, and naloxone pretreatment studies were 24.2 ± 5.1, 30.5 ± 8.2, and 22.9 ± 5.3 min, respectively. There was a statistically significant difference in the average clearance rate between retest and naloxone pretreatment studies in the visual cortex (*p* = 0.0143), whereas no statistical difference was observed among three studies in cerebellar cortex. These results indicate that there is a low level of MOR in the visual cortex that can be blocked by naloxone, whereas no blocking effect of naloxone was observed in the cerebellar cortex. Therefore, the cerebellar cortex is a better region of interest in NHPs for use in reference region-based PET data analyses such as SUVR or reference tissue-based kinetic modeling studies.

### Test-retest and naloxone blocking studies in the CNS

**Fig. 2:**
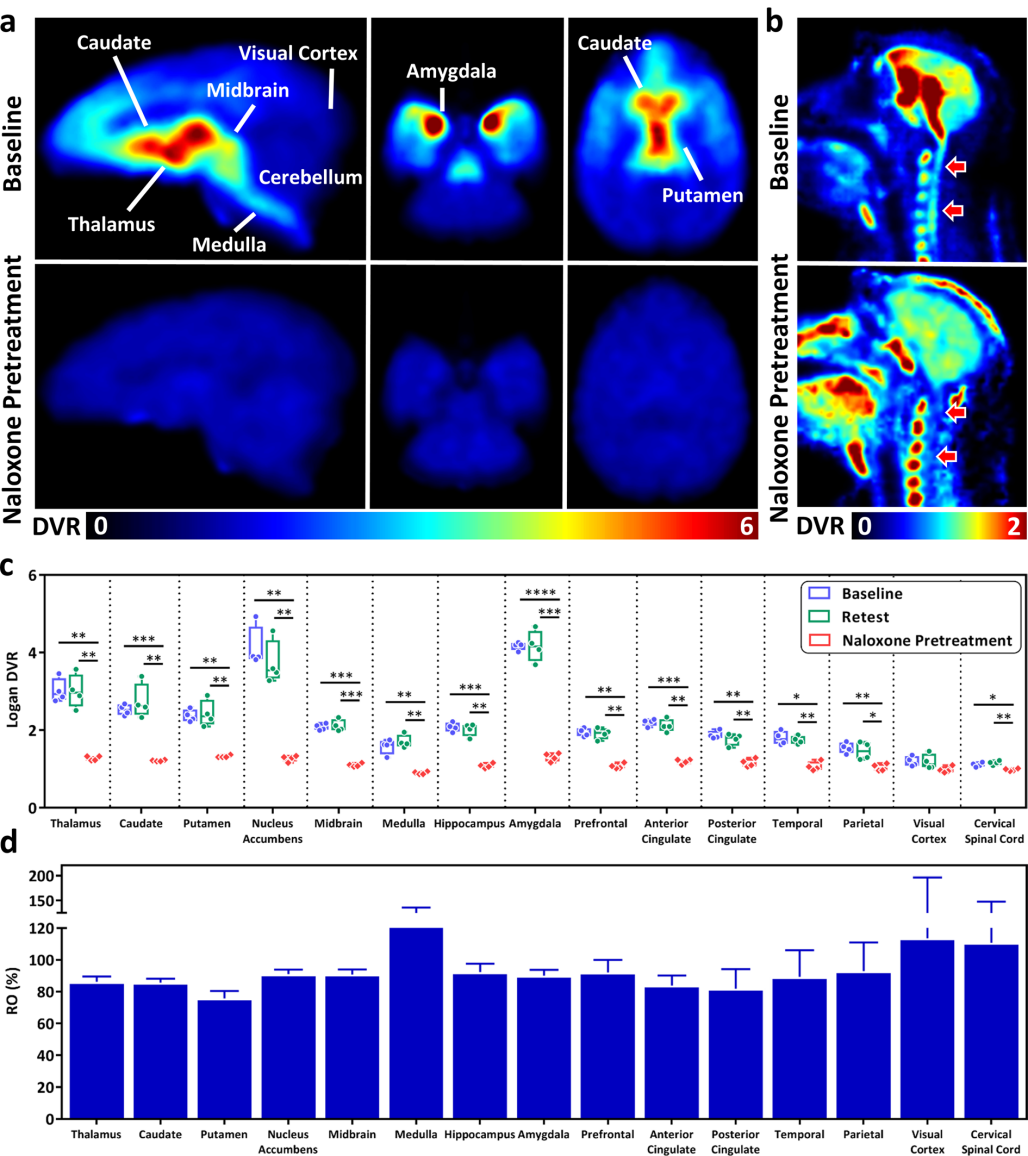
Naloxone blockade via IM administration 10 min prior to [^11^C]CFN injection. Representative Logan DVR images of the same NHP in baseline and naloxone pretreatment studies in (**a**) brain and (**b**) cervical spinal cord. (**c**) Box plot of Logan DVR for baseline, retest, and naloxone pretreatment, and (**d**) bar graph of %RO (mean ± standard deviation) in the different brain regions and cervical spinal cord. Statistical significance of p value calculated by paired t-test: **** *p* < 0.0001, *** *p* < 0.001, ** *p* < 0.01, and * *p* < 0.05

Figure 2a and b display the representative Logan DVR images of a baseline and the corresponding naloxone pretreatment scans in the brain and cervical spinal cord. The brain distribution of [^11^C]CFN in the baseline study (Fig. 2a and c) showed high radiotracer uptake in regions expressing the MOR, including thalamus, caudate, putamen, nucleus accumbens, amygdala, hippocampus, and midbrain. A lower DVR was observed in the cerebral cortices, medulla, and cervical spinal cord (Fig. 2b and c). There were no significant differences in the TACs of baseline and retest studies in regions of the CNS (Supplementary Fig. 1). The %Var of DVRs in most of the CNS regions was below 10% (Supplementary Table 2). A higher test-retest variability was observed in the nucleus accumbens (19.3 ± 8.7%) and medulla (13.3 ± 18.2%). There were no statistically-significant differences in the DVRs between baseline and retest studies in all regions of the CNS regions (Fig. 2c, and Supplementary Table 2).

In the naloxone pretreatment studies (IM administration), a significant blocking effect was observed in most of the brain regions as well as in cervical spinal cord (Fig. 2a-c, Supplementary Fig. 1, and Supplementary Table 2). The visual cortex was the only region showing a trend of naloxone blockade (baseline DVR = 1.2 ± 0.1 and naloxone pretreatment DVR = 1.0 ± 0.1) that did not reach statistical significance (Fig. 2c). The %RO of the naloxone blocking was 80 to 93% in most of the MOR abundant regions (Fig. 2d and Supplementary Table 2), and a slightly lower %RO was observed in putamen (75.8 ± 4.7%). A higher than 100% RO was observed in the small VOIs (i.e., medulla and cervical spinal cord) and brain regions with a low density of MOR such as the visual cortex, due to the calculated DVR being lower than 1.0 in the naloxone pretreatment studies.

### Displacement studies of [^11^C]CFN by naloxone and GSK1521498 in CNS

**Fig. 3:**
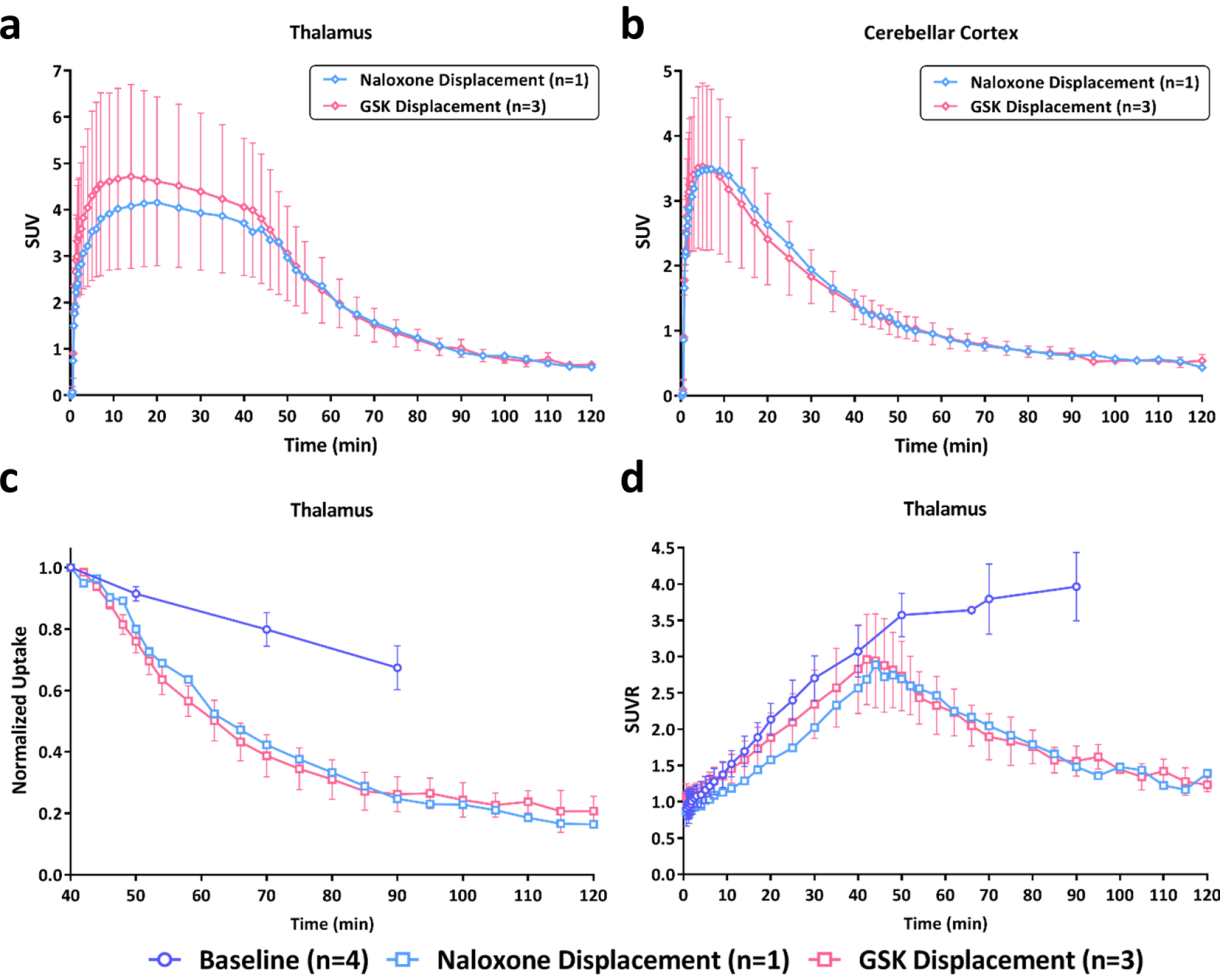
TACs of naloxone or GSK1521498 (GSK) displacement in (**a**) thalamus and (**b**) cerebellar cortex. Displacement rate of naloxone and GSK1521498 in comparison with thalamus TACs of (**c**) normalized uptake and (**d**) SUVR for displacement and baseline studies. Data points present as mean ± standard deviation

To characterize the ability of naloxone and GSK1521498 to compete with [^11^C]CFN binding to the MOR, a displacement study was conducted. In this study, IV administration of naloxone or GSK1521498 was given at 40 min post [^11^C]CFN injection, which represents the time point when [^11^C]CFN begins to reach a “plateau phase” (Supplementary Fig. 2). The TACs of representative CNS regions for the displacement studies are shown in Fig. 3a and Supplementary Fig. 3. Both naloxone and GSK1521498 enhanced the rate of washout of [^11^C]CFN in the MOR-abundant brain regions (and cervical spinal cord) and reduced it to the level of cerebellar cortex (reference region) by 90 min (i.e., 50 min after injection of the drug). There was no detectable decrease of [^11^C]CFN binding in cerebellar cortex following the IV administration of naloxone or GSK1521498 (Fig. 3b).

In order to compare the effect of naloxone and GSK1521498 on enhancing the rate of washout of [^11^C]CFN (i.e., prevent rebinding the radiotracer to the MOR), the normalized TACs of post-displacement phase (40–120 min) was plotted and shown in Fig. 3c and Supplementary Fig. 4. The ability of naloxone and GSK1521498 to prevent the [^11^C]CFN rebinding to the MOR is similar, and no differences were observed in the rate of “displacement” of [^11^C]CFN between GSK1521498 and naloxone (Fig. 3c). The clearance half-times of [^11^C]CFN post-IV injection of GSK1521498 or naloxone in thalamus were 22.3 ± 3.4 min and 23.8 min respectively. In the cervical spinal cord, the clearance half-times of [^11^C]CFN was greater than 80 min in all of the displacement studies (Supplementary Fig. 4). The SUVRs of [^11^C]CFN began to decrease at approximately 4–6 min after naloxone or GSK1521498 challenge and diminished to near unity (full displacement of radiotracer) at 70–80 min in all the brain regions (Fig. 3d and Supplementary Fig. 5). That is, [^11^C]CFN displacement by naloxone and GSK1521498 occurred with the same temporal pattern.

**Fig. 4:**
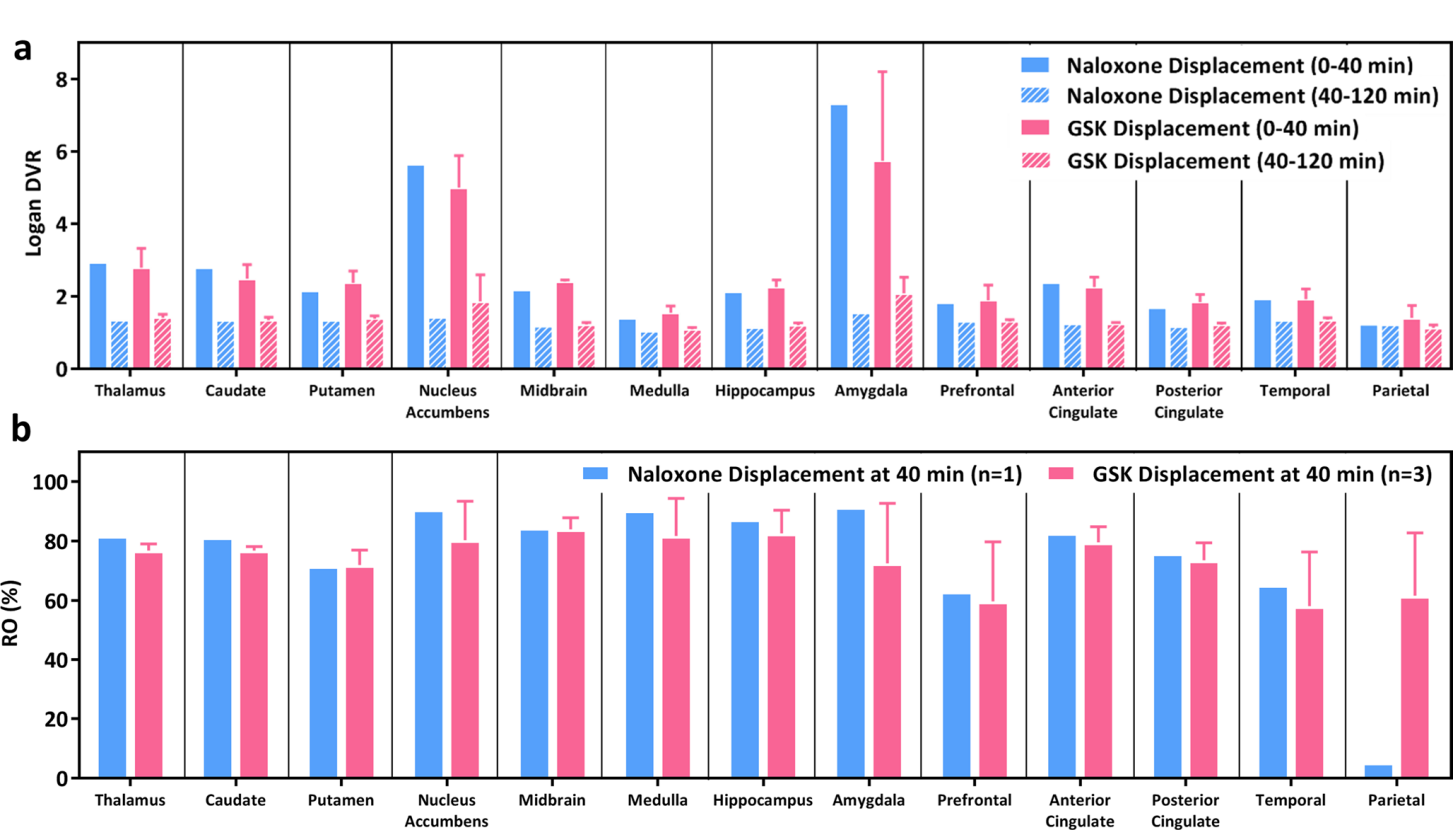
Naloxone or GSK1521498 (GSK) blockade via IV administration 40 min post [^11^C]CFN injection. (**a**) Logan DVR and (**b**) %RO in different brain regions. Data present as mean ± standard deviation

The %RO of naloxone and GSK1521498 in the displacement studies was calculated by comparing the 0–40 min DVR (prior to IV administration of naloxone and GSK1521498) and 40–120 min DVR (post-IV administration of naloxone and GSK1521498; Fig. 4) in the same [^11^C]CFN imaging study. The 40–120 min DVRs were significantly lower in most of the brain regions for all the displacement studies (Fig. 4a), revealing a significant displacement effect of naloxone and GSK1521498 challenge (~ 70–90% RO) in the MOR abundant brain regions, and an approximately 60% RO in brain regions with a low density of MOR (Fig. 4b).

### [^11^C]CFN uptake in peripheral organs

**Fig. 5:**
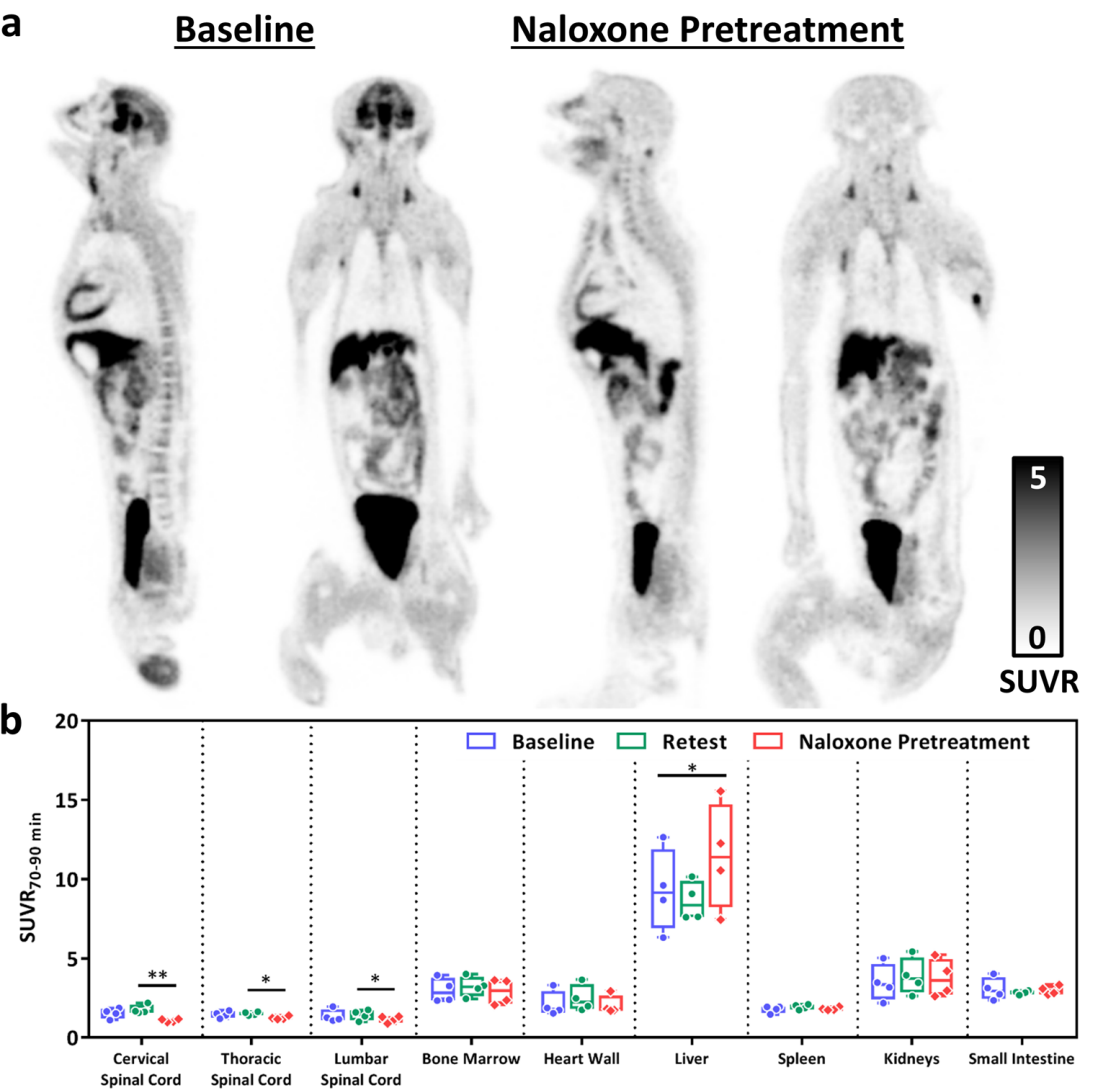
(**a**) Representative total-body SUVR_70 − 90 min_ images of the same NHP in baseline and naloxone pretreatment studies. (**b**) Box plot of SUVR_70 − 90 min_ for baseline, retest, and naloxone pretreatment studies in the spinal cord and peripheral organs. Statistical significance of p value calculated by paired t-test: ** *p* < 0.01, * *p* < 0.05

The TACs of baseline, retest, and naloxone pretreatment studies for peripheral organs are shown in Supplementary Fig. 6. The uptake of [^11^C]CFN reached a plateau phase at approximately 50 min and remained steady for the remainder of the scan (Supplementary Fig. 6). There is no difference among the TACs for the baseline, retest, and naloxone pretreatment studies in the skeletal muscle; it has been reported that the MOR expression in skeletal muscle is low [15]. Therefore, skeletal muscle was used as the reference region to calculate SUVR_70 − 90 min_ to compare the test-retest variability and the effect of naloxone pretreatment on tracer uptake in the spinal cord and peripheral organs. There is no significant difference between baseline and retest SUVR_70 − 90 min_ in spinal cord and peripheral organs (Fig. 5b and Supplementary Table 3). The %Var of the test-retest variability in the spinal cord, spinal bone marrow, heart wall, spleen, and small intestine were in the range of 8–16% (Supplementary Table 3). A higher test-retest variability (%Var > 20%; Supplementary Table 3) was observed in liver and kidneys.

In the naloxone pretreatment studies (Fig. 5 and Supplementary Table 3), there was a significant effect of naloxone on radiotracer binding in cervical (*p* = 0.0082), thoracic (*p* = 0.0133) and lumbar (*p* = 0.0222) spinal cord. Although naloxone lowered the mean uptake of [^11^C]CFN in spinal bone marrow (3–10% decrease in blocking study) and heart wall (1–15% decrease in blocking study), the difference was not statistically significant.

**Fig. 6:**
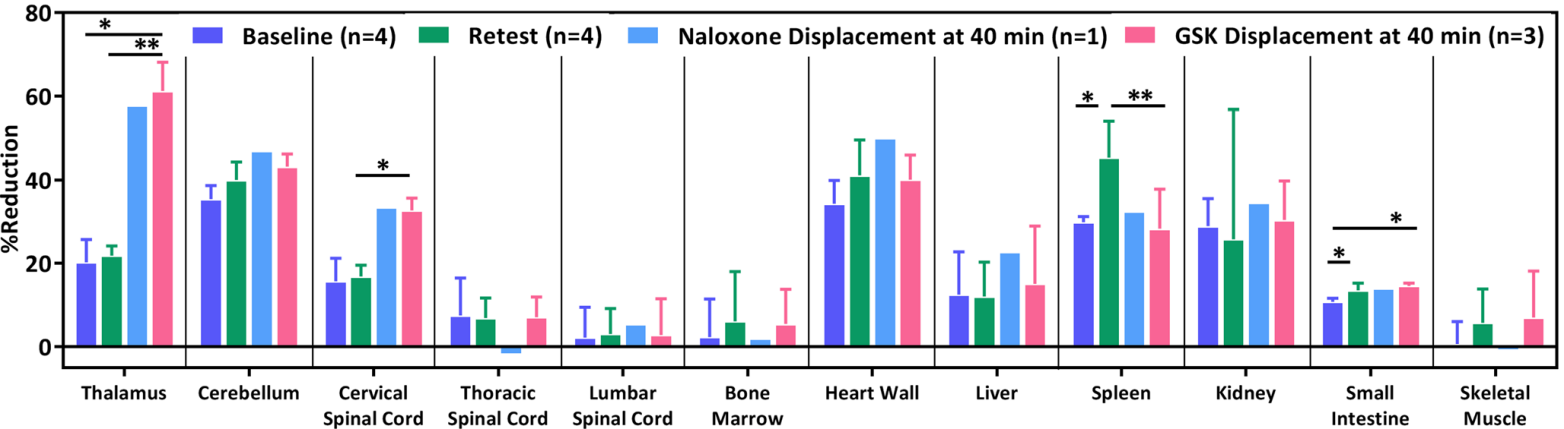
%Reduction between 40- and 70-min SUV for 2 representative brain regions, spinal cord, and peripheral organs. Data presented as mean ± standard deviation. Statistical significance of p value calculated by paired t-test: ** *p* < 0.01, * *p* < 0.05

To determine the displacement effect of naloxone and GSK1521498 in both CNS and peripheral organs, the %Reduction between 40- and 70-min SUV (prior and post naloxone or GSK1521498 challenge) within the same scans were calculated for the baseline and displacement studies (Fig. 6). As compared to the baseline/retest study, thalamus and cervical spinal cord were observed to have a higher %Reduction in both naloxone and GSK1521498 displacement studies. The binding of [^11^C]CFN was slightly diminished by naloxone in heart wall (Fig. 6 and Supplementary Fig. 7). No decline of [^11^C]CFN uptakes were observed after naloxone or GSK1521498 challenge in thoracic and lumbar spinal cord, spinal bone marrow, liver, spleen, kidneys and skeletal muscles (Supplementary Fig. 7).

## Discussion

This study demonstrated the total-body imaging and test-retest variability of the MOR agonist, [^11^C]CFN. Blocking studies were also conducted using the MOR antagonists, naloxone and GSK1521498, which were given via IM or IV administration in rhesus macaques. The %Var of the test-retest measures across brain regions for DVR of [^11^C]CFN was approximately 8%, which is consistent with the previous test-retest study in humans [18], demonstrating the high reproducibility of [^11^C]CFN in imaging brain MOR. Taking advantage of the high sensitivity total-body scanner, PennPET Explorer, [^11^C]CFN uptake in spinal cord and peripheral organs was also measured. A high reproducibility of [^11^C]CFN DVR in cervical spinal cord was also observed (~ 6% test-retest variability). Due to the low perfusion and uptake of [^11^C]CFN in the thoracic (SUV at peak = 1.4 ) and lumbar (SUV at peak = 1.2 ) spinal cord, the semi-quantitative parameter, SUVR_70 − 90 min_, was used to evaluate the reproducibility of [^11^C]CFN; this showed a somewhat higher test-retest variability than what was observed in brain. The %Var of test-retest was also higher in peripheral organs than in the CNS, which may be due to either the mixed signal of the parent compound and radiolabeled metabolites, and the variability of the biological clearance of [^11^C]CFN and its radiolabeled metabolites among the NHPs.

Logan graphical analysis using the occipital cortex as the reference region has been widely used in quantitative [^11^C]CFN brain imaging studies in human [18–21], NHP [23, 24] and porcine [31]. In the current study, although the occipital/visual cortex showed low uptake of [^11^C]CFN in the baseline scan, there was a notable decrease in [^11^C]CFN uptake in the naloxone pretreatment study, and the naloxone or GSK1521498 displacement studies (Supplementary Fig. 8). In contrast, the cerebellar cortex showed the lowest [^11^C]CFN uptake in the baseline study and no detectable effects of naloxone or GSK1521498 in both the blocking and displacement studies. It has been reported that there is no significant binding of MOR in a [^35^S]GTPγS autoradiography study in cerebellum of cynomolgus monkey brain [32]. These results indicate that the cerebellar cortex may serve as a better reference region for quantitative [^11^C]CFN brain imaging studies in NHPs.

A study was also done to compare the route of administration on %RO of naloxone at MOR using [^11^C]CFN. There was no difference in the %RO of the same dose of naloxone in MOR abundant brain regions when naloxone was given either via IM or IV injection (Supplementary Table 4). For instance, the %RO in thalamus was 87.9% and 88.1% for IM and IV administration respectively in NHP-3. Saccone et al. [23]. has reported that the RO in thalamus is slightly greater in the same dose of naloxone given by IV versus intranasal administration in rhesus monkeys, whereas no difference in RO in thalamus was observed via IM and IV administration in a follow-up study [24].The later study is consistent with our results. In the current study, IM administration of naloxone (0.14 mg/kg), the average %RO in thalamus and striatum was 86% and 81% respectively. This is higher than in the previous report from Scott et al. [24], who observed a %RO of approximately 65% and 74% in thalamus and basal ganglia for the same dose of naloxone given by the same injection route. The difference in %RO might be attributed to the choice of the reference region (i.e., cerebellar cortex versus occipital cortex) for DVR calculation. By using occipital/visual cortex as reference region to calculate DVR in the current study, the average %RO in thalamus and striatum was 80% and 70% respectively (Supplementary Fig. 9). It should also be noted that gender differences in [^11^C]CFN binding to MOR in human brain have been reported [17]. Hence, the differences of RO in NHP brain between the two studies may also due to the gender differences, since male rhesus monkeys were used in the current study and female rhesus monkeys were used in the previous study [24].

The function of displacement studies with a PET radiotracer is to measure the ability of an antagonist to compete with the radiotracer for binding to a CNS receptor. This is measured by the increased rate of washout from a region of interest following administration of a displacer, which prevents the rebinding of the radiotracer to its target receptor. In the naloxone displacement studies, the SUVRs of [^11^C]CFN in the CNS began to decrease at the 4–6 min time frame post naloxone IV administration. This indicates that it takes naloxone approximately 4–6 min to reach concentrations in the CNS that prevents the rebinding of [^11^C]CFN to the MOR. In addition, this result of the naloxone displacement studies is similar to the clinical reports suggesting that an average of 6–8 min response time is required for naloxone to reverse opiate overdose when given by IM administration [33–35]. It is worth noting that the time of naloxone to affect the binding of opioids for MORs may differ depending on the route of administration.

In the studies comparing the pharmacokinetics of naloxone and GSK1521498 for [^11^C]CFN displacement, there were no differences in the shape of TACs, %RO, displacement rate, and the response time for displacing [^11^C]CFN from MOR in the brain and cervical spinal cord. These results indicate that the behavior of the MOR selective antagonist/inverse agonist GSK1521498 is similar to naloxone for MOR target engagement in vivo, and suggests that GSK1521498 may be a potential alternative to naloxone for opioid overdose rescue. However, more in vivo studies are needed, such as dose-response relationships and route of administration of the drug, in order to evaluate the capability of GSK1521498 to reverse opiate overdose.

Previous studies have shown that the MOR is expressed in the dorsal horn of lumber spinal cord in rats [16, 36], and a moderate level of MOR expression has been reported in human spinal cord [15]. Our results revealed a low level of specific binding of [^11^C]CFN in baseline/retest studies in the cervical spinal cord (average DVR = 1.1), and demonstrated a significant blocking effect of MOR antagonists, naloxone and GSK1521498. In the thoracic and lumber spinal cord, there is a hint of naloxone blockade in the SUVR_70 − 90 min_ in the pretreatment versus baseline/retest studies. However, there was no displacement of [^11^C]CFN following IV administration of naloxone or GSK1521498. These data suggest that [^11^C]CFN may be feasible for imaging MOR density in the cervical spinal cord, but not sufficient for imaging the thoracic and lumber spinal cord due to the low radiotracer uptake. This is the first study demonstrating the ability to image MORs in spinal cord in vivo with PET.

Mixed results have been reported on the expression of MOR in human heart. For example, Peng et al. [15]. reported no MOR expression in heart tissue by using absolute quantitative real-time reverse transcriptase PCR to quantitate MOR mRNA. However, in a later study, Sobanski et al. [37] used immunohistochemical techniques to demonstrate MOR expression in myocardial cells present in the heart wall of the left ventricle. An earlier PET imaging study demonstrated a 25% reduction of [^11^C]CFN binding potential in human heart when naloxone was given in a dose of 0.2 mg/kg via IV administration 5 min prior to injection of the radiotracer [26]. These results suggest the potential of [^11^C]CFN to image cardiac MORs in vivo. In our study, the results of naloxone blockade in the pretreatment study were mixed. In comparison of the [^11^C]CFN SUVR_70 − 90 min_ to the two control scans (baseline and retest) (Supplementary Fig. 10a), an average of 17% reduction of [^11^C]CFN SUVR_70 − 90 min_ was observed in 2 NHPs, a 4% increase of [^11^C]CFN SUVR_70 − 90 min_ was observed in one NHP, and mixed results were observed in another NHP. In the displacement study, there was gradual change of the TACs after naloxone or GSK1521498 administration (Supplementary Fig. 10b). These results indicate that the dose of 0.14 mg/kg naloxone used in the current study may not be high enough to block the binding of [^11^C]CFN to cardiac MORs. Hence, more studies are needed to evaluate the feasibility of imaging cardiac MORs by PET in vivo.

It has been reported that there is low density of MORs expressed in small intestines [15, 16]. However, there was no effect of naloxone or GSK1521498 in the blocking or displacement studies on the uptake of [^11^C]CFN in the current study. This may be due to the mixed signal of [^11^C]CFN and radiolabeled metabolites in the small intestines, since [^11^C]CFN is metabolized in the liver and excreted via the small intestines.

In conclusion, specific uptake of [^11^C]CFN was observed in the MOR-abundant regions in both CNS and peripheral organs under baseline conditions. The blocking effect of both naloxone and GSK1521498 under pretreatment or displacement conditions were also observed in MOR-abundant regions including brain and cervical spinal cord. It took approximately 4–6 min for naloxone or GSK1521498 to distribute to CNS and displace the binding of [^11^C]CFN. The pharmacokinetic behavior of GSK1521498 and naloxone displacement were similar, suggesting that GSK1521498 could be a potential alternative to naloxone for reversing opioid overdose. In peripheral organs, only heart wall showed a hint of naloxone or GSK1521498 blockade.

By using the high sensitivity total-body imager, PennPET Explorer, in this study, we were able to image MORs in the entire CNS (i.e., brain and spinal cord). In addition to imaging MORs in the CNS, our study demonstrated the capability to image MORs in the cardiovascular system and peripheral organs (e.g., spleen) in vivo. The use of total body PET in combination with the blocking and displacement studies revealed the potential to study the dynamic interactions between a MOR agonist (i.e., [^11^C]CFN) and MOR antagonists (i.e., naloxone and GSK1521498) across the brain, spinal cord and peripheral organs in vivo. These data indicate that [^11^C]CFN total-body PET scans could provide a novel approach for studying mechanism of action of MOR drugs used in the treatment of acute and chronic opioid use disorder, and the effect of chronic opioid use disorder on the expression on MOR in the brain and spinal cord.

### Electronic supplementary material

Below is the link to the electronic supplementary material.

Supplementary Material 1

## Data Availability

The article contains complete data used to support the findings of this study.
