# Supplementary material for: Total-body imaging of mu-opioid receptors with [11C]carfentanil in non-human primates
Source: Eur J Nucl Med Mol Imaging. 2024 May 9;51(11):3273–83. doi: 10.1007/s00259-024-06746-2 (PMC11368985; doi:10.1007/s00259-024-06746-2)
Supplement: Supplementary file 1 — Supplementary Material 1 [file 259_2024_6746_MOESM1_ESM.docx]

Total-body Mu-opioid Receptors [^11^C]Carfentanil Study in Non-human Primates

Chia-Ju Hsieh^1^, Catherine Hou^1^, Hsiaoju Lee^1^, Cosette Tomita^1^, Alexander Schmitz^1^, Konstantinos Plakas^1^, Jacob Dubroff^1^, and Robert H. Mach^1*^

^1^ Department of Radiology, Perelman School of Medicine, University of Pennsylvania, Philadelphia, Pennsylvania 19104, United States

*Corresponding Author. E-mail: [rmach@pennmedicine.upenn.edu](http://rmach@pennmedicine.upenn.edu)

**Supplementary Information**

Characterization QC test of [^11^C]CFN 1

Characterization of GSK1521498 2

Supplementary Table 1. Study metrics of [^11^C]CFN scans 3

Supplementary Fig. 1. TACs of SUV in different brain regions and cervical spinal cord. 4

Supplementary Table 2. Test-retest variability and %RO of naloxone pretreatment studies for brain and cervical spinal cord 5

Supplementary Fig. 2. TACs of baseline SUVR in different brain regions. 6

Supplementary Fig. 3. TACs of naloxone or GSK1521498 (GSK) displacement in different brain regions and cervical spinal cord. 7

Supplementary Fig. 4. Displacement rate of naloxone and GSK1521498 in comparison with TACs of normalized uptake in different brain regions and cervical spinal cord. 8

Supplementary Fig. 5. Displacement rate of naloxone and GSK1521498 in comparison with TACs of SUVR in different brain regions and spinal cord. 9

Supplementary Table 3. Test-retest variability and %Diff_SUVR_ of naloxone pretreatment studies for spinal cord and peripheral organs 10

Supplementary Fig. 6. TACs of SUV for spinal cord and peripheral organs 11

Supplementary Fig. 7. TACs of naloxone or GSK1521498 (GSK) displacement for spinal cord and peripheral organs. 12

Supplementary Fig. 8. TACs of naloxone or GSK1521498 (GSK) displacement for visual cortex. 13

Supplementary Fig. 9. Logan DVR by using occipital as reference region for baseline and naloxone pretreatment. 14

Supplementary Table 4. %RO of naloxone pretreatment and displacement studies for NHP-3 15

Supplementary Fig. 10. Heart wall individual TACs and SUVR_70-90min_. 16

Supplementary QC test of [^11^C]CFN

The ultra performance liquid chromatography (UPLC) method used for the analysis was consistent with the guidelines as per USP <823> positron emission tomography drugs for compounding, investigational, and research uses and USP <621> chromatography. UPLC testing determines the radiochemical and chemical purity of the final drug product and also the radiochemical identity of the final drug product. The UPLC method used was validated for the specificity, linearity, accuracy, precision, and suitability for the determination of radiochemical purity and identity for batch release. A calibration curve was generated to establish the linear relationship of the drug mass and UV absorbance. The mass of drug substance from production was calculated by using the existing calibration curve. The mass of total impurities from production was calculated using the same method, assuming the conversion factor of one.

A Waters Acquity H class was used for the sample analysis. The analysis mobile phase was composed of 40% of 0.1 M ammonium formate and 60% of methanol. The column was a Waters BEH, C18, 1.7 µM, 2.1 x 50 mm or equivalent. The flow rate was 0.5 mL/min, and UV detector was set to 209 nm. In this condition, the precursor was eluted at around 0.4 min while carfentanil was eluted at around 1.2. min. The total analyses time was 5 min for each injection. No carry-over was found in this condition. The limit of detection (LOD) and limit of quantification (LOQ) in the systems that were tested were 0.02/0.05 and 0.1/0.2 ug/mL, respectively, for the two systems qualified. We had minimal concern of the existence of precursor in the final preparation as demonstrated in the analyses results shown in the later sections.

After drug manufacture, the analytical system was injected with a sample of the final drug product (Mass injection) and a sample of final drug product and reference standard mixture (radiochemical identity injection, ID injection). Radiochemical purity analysis was performed by comparing the area of [^11^C]carfentanil peak to the sum of all radioactivity peaks from ID injection. Radiochemical identity was analyzed by comparing the retention times of the product to that of the reference standard from the ID injection.

Characterization of GSK1521498

The multistep synthesis of GSK1521498 was accomplished using published methods.  ^1^H-NMR (DMSO) d 9.97 (d, 2H), 8.67 (s, 1H), 8.49 (s, 1H), 8.15 (d, 1H, J = 7.8 Hz), 7.91 (d, 1H J = 8.3 Hz), 7.73 (d, 2H, J = 8.9 Hz), 7.66 (t, 1H, J = 7.8 Hz), 7.30-7.27 (m, 2H), 7.23-7.20 (m, 2H), 4.32 (s, 2H), 4.16 (q, 1H, J = 6.8 Hz), 3.40 (dd, 2H, J = 8.1 Hz), 3.25 (dd, 2H, J = 7.8 Hz).

**Supplementary Table 1.** Study metrics of [^11^C]CFN scans

| Studies | NHP-1 | NHP-2 | NHP-3 | NHP-4 |
| --- | --- | --- | --- | --- |
| Baseline | v | v | v | v |
| Retest | v | v | v | v |
| Naloxone Pretreatment **(**0.14 mg/kg, IM administration**)** | v | v | v | v |
| Naloxone Displacement **(**0.14 mg/kg, IV administration**)** |  |  | v |  |
| GSK1521498 Displacement **(**0.14 mg/kg, IV administration**)** |  | v | v | v |


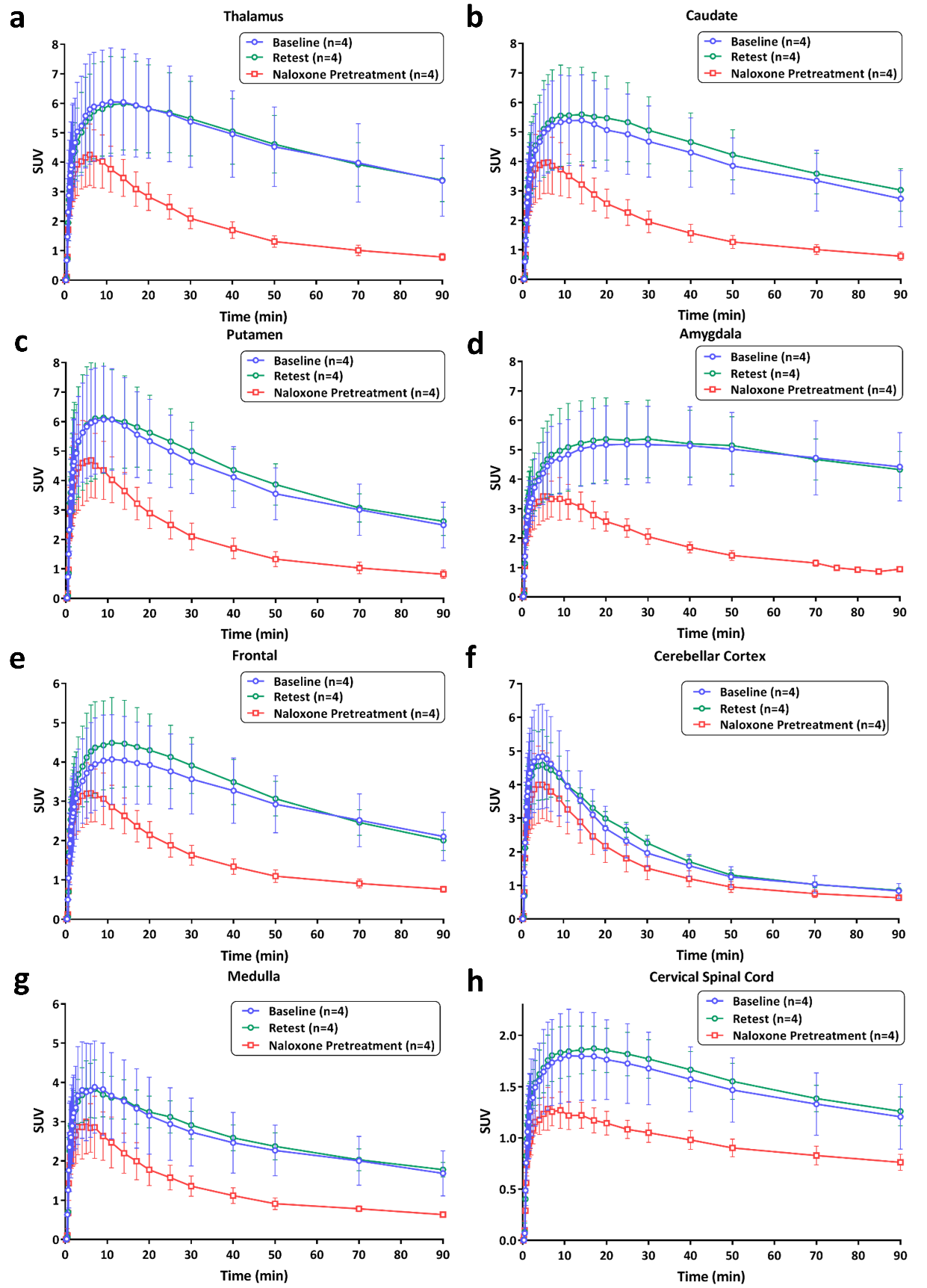


Supplementary Fig. 1. TACs of SUV in different brain regions and cervical spinal cord. (**a**) thalamus, (**b**) caudate, (**c**) putamen, (**d**) amygdala, (**e**) frontal, (**f**) cerebellar cortex, (**g**) medulla, and (**h**) cervical spinal cord in baseline, retest, and naloxone pretreatment studies. Data points present as mean ± standard deviation.

**Supplementary Table 2.** Test-retest variability and %RO of naloxone pretreatment studies for brain and cervical spinal cord

|  | Logan DVR | | | %Var | %RO | |
| --- | --- | --- | --- | --- | --- | --- |
| Region | Baseline | Retest | Naloxone Pretreatment | Test-retest | vs. Baseline | vs. Retest |
| Thalamus | 3.0 ± 0.3 | 3.0 ± 0.4 | 1.3 ± 0.1 | 3.8 ± 3.9 | 86.2 ± 3.4 | 85.7 ± 5.0 |
| Caudate | 2.5 ± 0.1 | 2.7 ± 0.5 | 1.2 ± 0.0 | 9.9 ± 11.5 | 85.8 ± 2.4 | 87.1 ± 3.8 |
| Putamen | 2.4 ± 0.2 | 2.4 ± 0.3 | 1.3 ± 0.0 | 10.0 ± 8.9 | 75.8 ± 4.7 | 75.9 ± 7.3 |
| Nucleus accumbens | 4.1 ± 0.5 | 3.7 ± 0.6 | 1.3 ± 0.1 | 19.3 ± 8.7 | 91.1 ± 2.8 | 89.9 ± 2.4 |
| Midbrain | 2.1 ± 0.1 | 2.1 ± 0.2 | 1.1 ± 0.0 | 1.8 ± 3.1 | 90.8 ± 3.3 | 91.1 ± 2.7 |
| Medulla | 1.6 ± 0.2 | 1.7 ± 0.2 | 0.9 ± 0.0 | 13.3 ± 18.2 | 121.0 ± 15.0 | 114.5 ± 5.8 |
| Hippocampus | 2.1 ± 0.1 | 2.0 ± 0.2 | 1.1 ± 0.1 | 5.6 ± 3.8 | 92.3 ± 5.3 | 92.1 ± 5.0 |
| Amygdala | 4.2 ± 0.1 | 4.2 ± 0.4 | 1.3 ± 0.1 | 6.1 ± 5.9 | 90.2 ± 3.5 | 90.4 ± 2.7 |
| Prefrontal | 1.9 ± 0.1 | 1.9 ± 0.2 | 1.1 ± 0.1 | 8.3 ± 6.4 | 92.1 ± 7.9 | 91.6 ± 8.0 |
| Anterior cingulate | 2.2 ± 0.1 | 2.1 ± 0.2 | 1.2 ± 0.1 | 7.1 ± 5.6 | 83.8 ± 6.3 | 82.9 ± 5.4 |
| Posterior cingulate | 1.9 ± 0.1 | 1.8 ± 0.2 | 1.2 ± 0.1 | 6.2 ± 6.3 | 82.0 ± 12.2 | 79.4 ± 12.5 |
| Temporal | 1.8 ± 0.2 | 1.8 ± 0.1 | 1.1 ± 0.1 | 5.5 ± 3.7 | 89.4 ± 16.6 | 90.2 ± 14.6 |
| Parietal | 1.5 ± 0.1 | 1.5 ± 0.2 | 1.0 ± 0.1 | 9.1 ± 4.2 | 93.0 ± 17.9 | 88.5 ± 28.0 |
| Visual cortex | 1.2 ± 0.1 | 1.2 ± 0.2 | 1.0 ± 0.1 | 5.0 ± 2.1 | 113.5 ± 82.9 | 101.8 ± 179.8 |
| Cervical spinal cord | 1.1 ± 0.1 | 1.2 ± 0.1 | 1.0 ± 0.0 | 6.2 ± 3.4 | 110.8 ± 37.4 | 112.2 ± 19.4 |

**
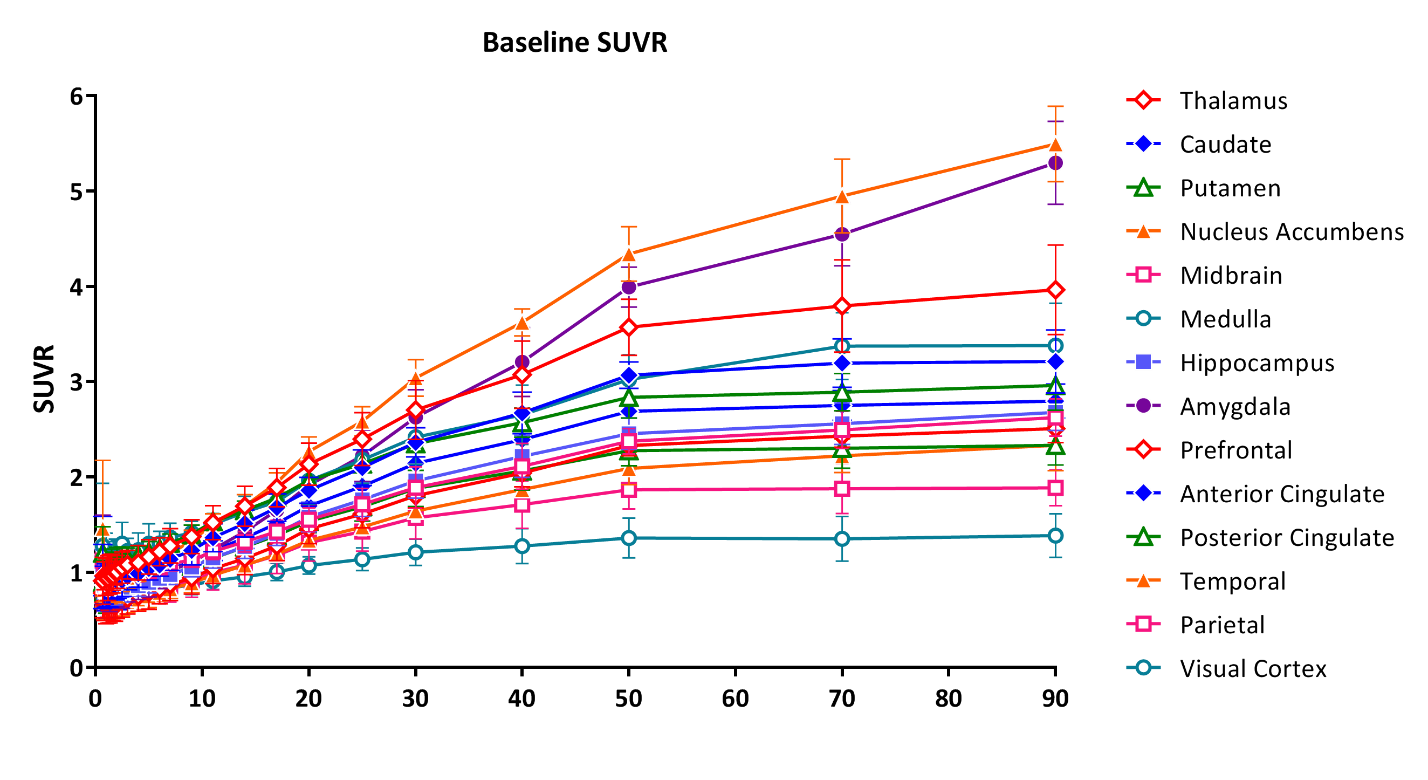
**

Supplementary Fig. 2. TACs of baseline SUVR in different brain regions. Data points present as mean ± standard deviation.


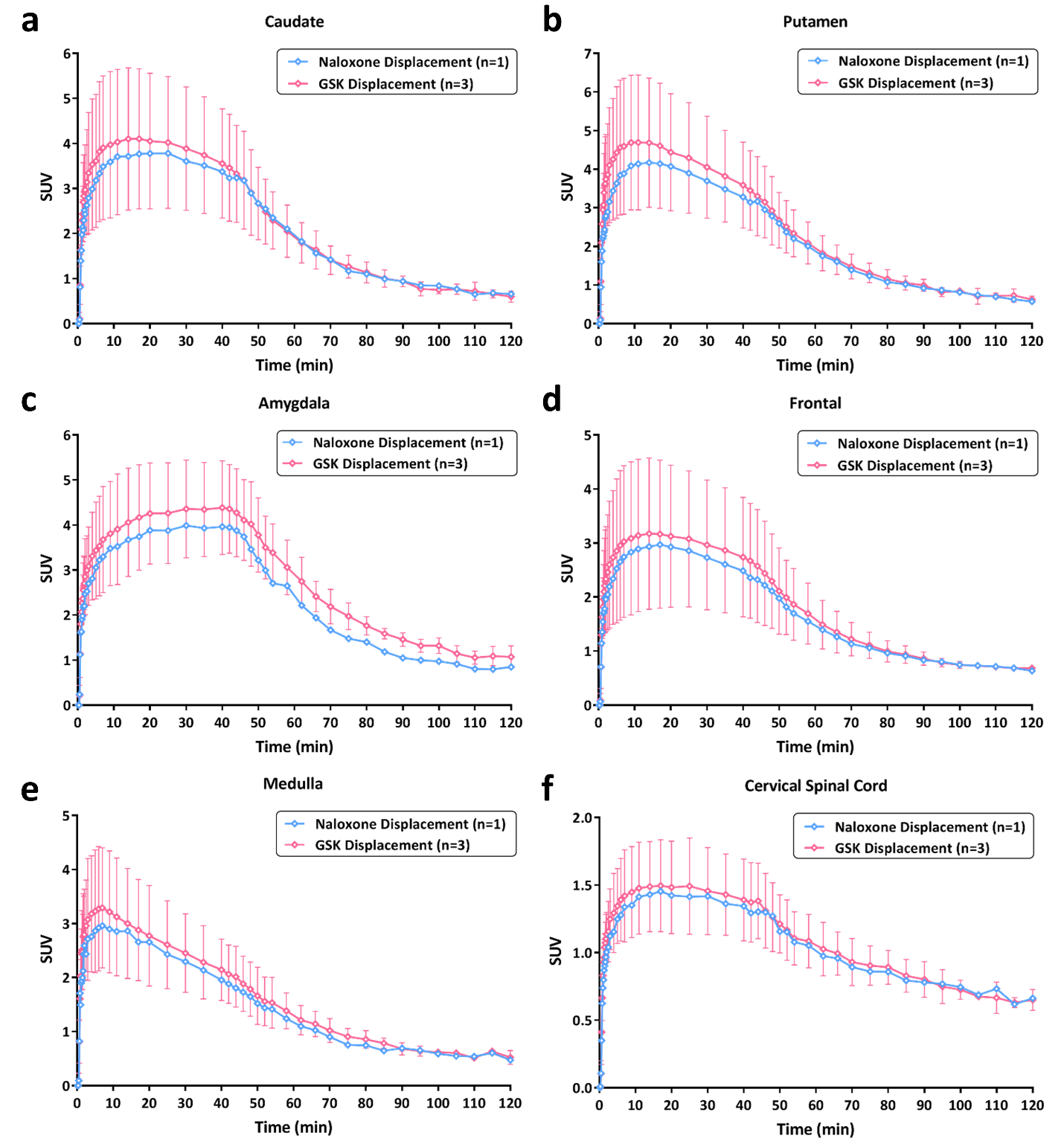


Supplementary Fig. 3. TACs of naloxone or GSK1521498 (GSK) displacement in different brain regions and cervical spinal cord. (**a**) caudate, (**b**) putamen, (**c**) amygdala, (**d**) frontal, (**e**) medulla, and (**f**) cervical spinal cord. Data points present as mean ± standard deviation.


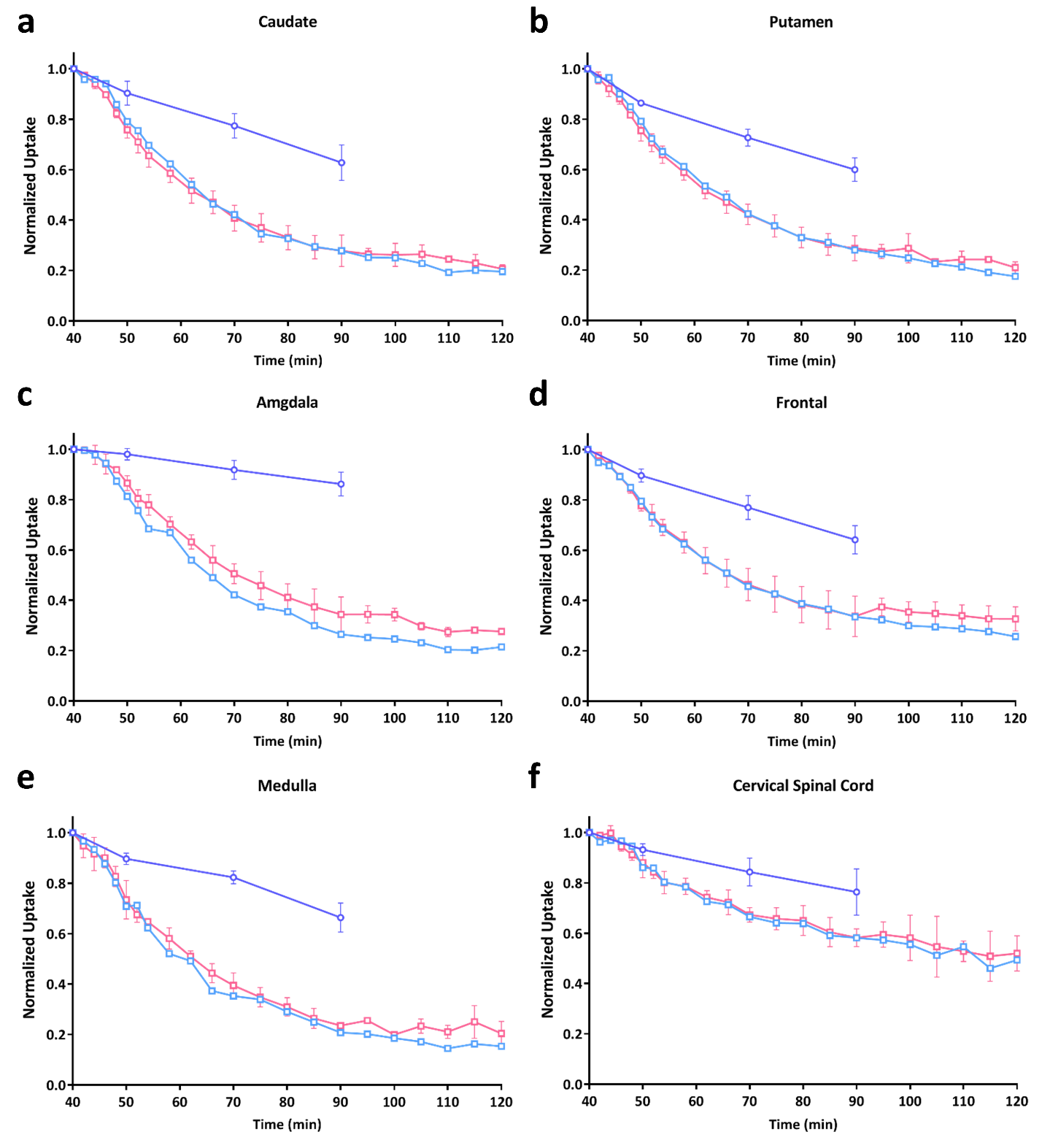


Supplementary Fig. 4. Displacement rate of naloxone and GSK1521498 in comparison with TACs of normalized uptake in different brain regions and cervical spinal cord. (**a**) caudate, (**b**) putamen, (**c**) amygdala, (**d**) frontal, (**e**) medulla, and (**f**) cervical spinal cord. Data points present as mean ± standard deviation.


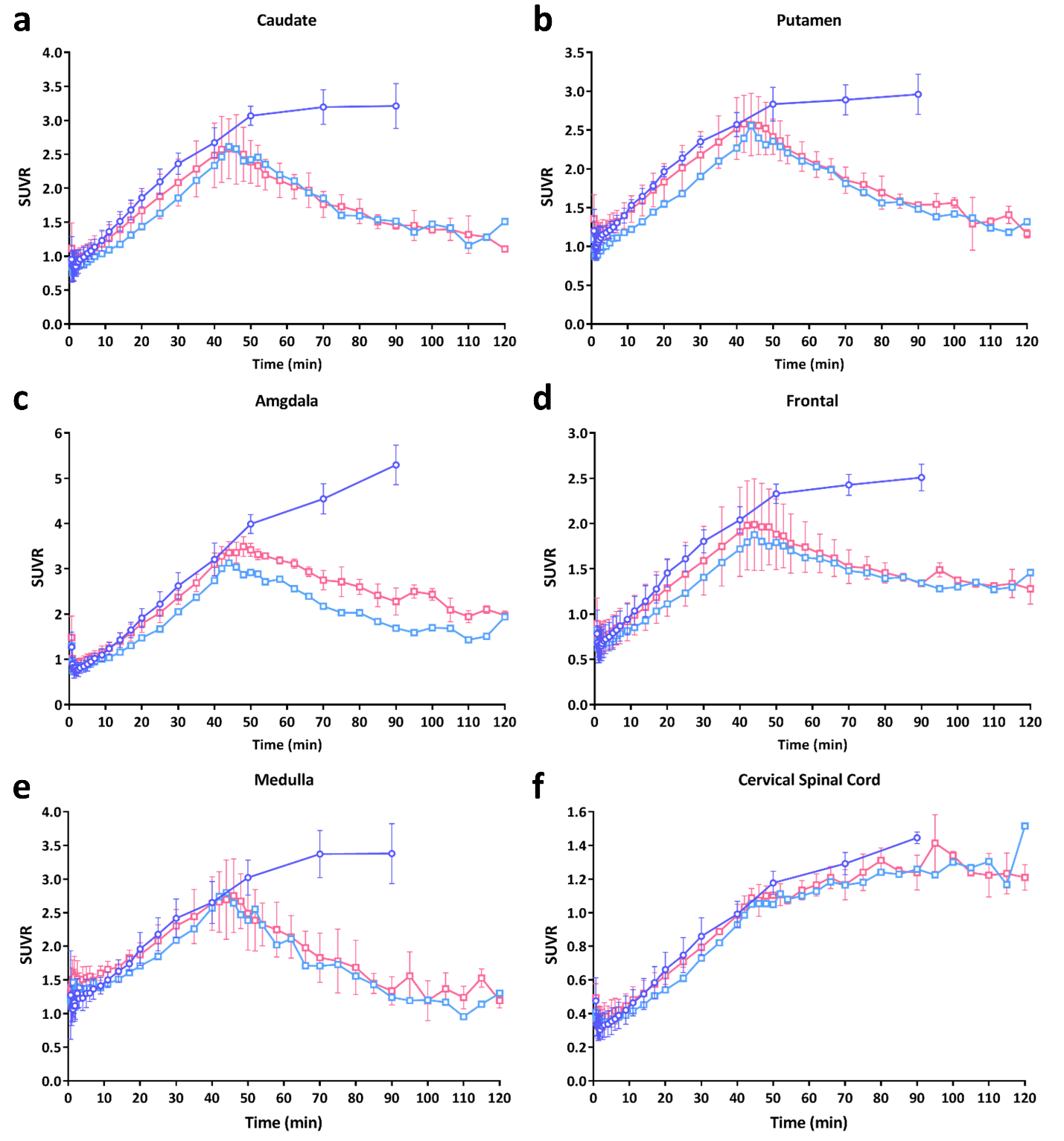


Supplementary Fig. 5. Displacement rate of naloxone and GSK1521498 in comparison with TACs of SUVR in different brain regions and spinal cord. (**a**) caudate, (**b**) putamen, (**c**) amygdala, (**d**) frontal, (**e**) medulla, and (**f**) cervical spinal cord. Data points present as mean ± standard deviation.

|  | SUVR_70-90min_ | | | %Var | %Diff_SUVR_ | |
| --- | --- | --- | --- | --- | --- | --- |
|  | Baseline | Retest | Naloxone Pretreatment | Test-retest | vs. Baseline | vs. Retest |
| Cervical Spinal Cord | 1.5 ± 0.3 | 1.8 ± 0.3 | 1.1 ± 0.1 | 15.1 ± 17.9 | 27.1 ± 20.7 | 38.3 ± 6.2 |
| Thoracic Spinal Cord | 1.5 ± 0.2 | 1.5 ± 0.1 | 1.2 ± 0.1 | 12.2 ± 12.6 | 12.1 ± 20.4 | 17.7 ± 6.0 |
| Lumbar Spinal Cord | 1.4 ± 0.4 | 1.4 ± 0.3 | 1.2 ± 0.2 | 13.9 ± 12.3 | 12.1 ± 18.5 | 18.0 ± 5.1 |
| Spinal Bone Marrow | 3.0 ± 0.8 | 3.2 ± 0.6 | 2.9 ± 0.8 | 8.0 ± 11.8 | 3.3 ± 9.5 | 10.3 ± 14.0 |
| Heart wall | 2.1 ± 0.8 | 2.5 ± 0.8 | 2.0 ± 0.6 | 16.2 ± 12.9 | 0.8 ± 9.8 | 15.2 ± 13.8 |
| Liver | 9.3 ± 2.6 | 8.6 ± 1.2 | 11.4 ± 3.4 | 26.1 ± 20.4 | -22.9 ± 13.2 | -37.7 ± 55.4 |
| Spleen | 1.8 ± 0.2 | 1.9 ± 0.2 | 1.8 ± 0.1 | 10.6 ± 6.9 | -2.6 ± 11.1 | 5.8 ± 9.9 |
| Kidneys | 3.5 ± 1.2 | 3.9 ± 1.2 | 3.8 ± 1.2 | 30.1 ± 12.1 | -10.4 ± 20.2 | -2.7 ± 39.1 |
| Small Intestine | 3.1 ± 0.7 | 2.8 ± 0.1 | 3.0 ± 0.3 | 14.6 ± 13.9 | -0.8 ± 21.7 | -5.2 ± 12.6 |

**Supplementary Table 3.** Test-retest variability and %Diff_SUVR_ of naloxone pretreatment studies for spinal cord and peripheral organs


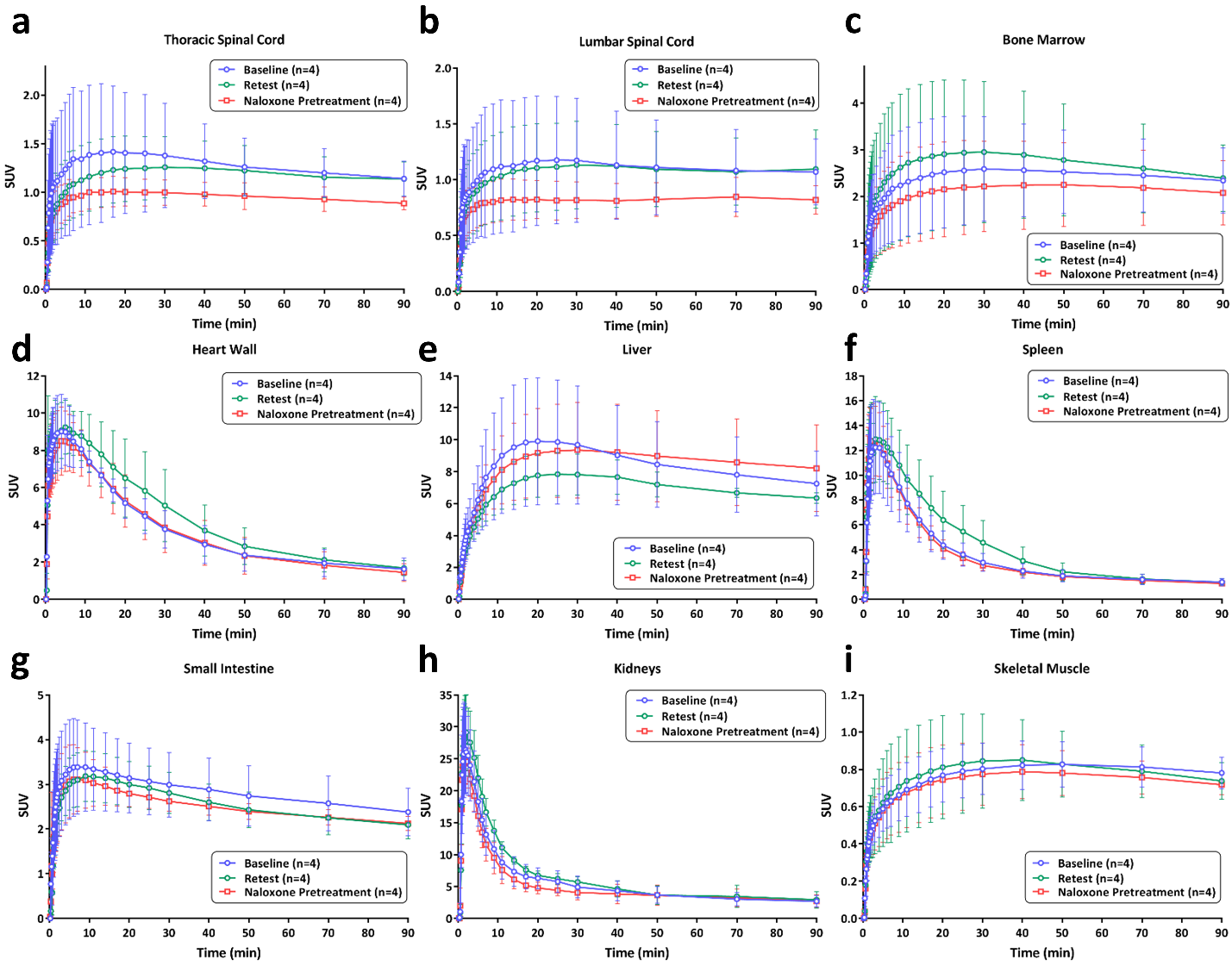


Supplementary Fig. 6. TACs of SUV for spinal cord and peripheral organs**.** (**a**) thoracic spinal cord, (**b**) lumbar spinal cord, (**c**) spinal bone marrow, (**d**) heart wall, (**e**) liver, (**f**) spleen, (**g**) small intestine, (**h**) kidneys, and (**i**) skeletal muscle in baseline, retest, and naloxone pretreatment studies. Data points present as mean ± standard deviation.


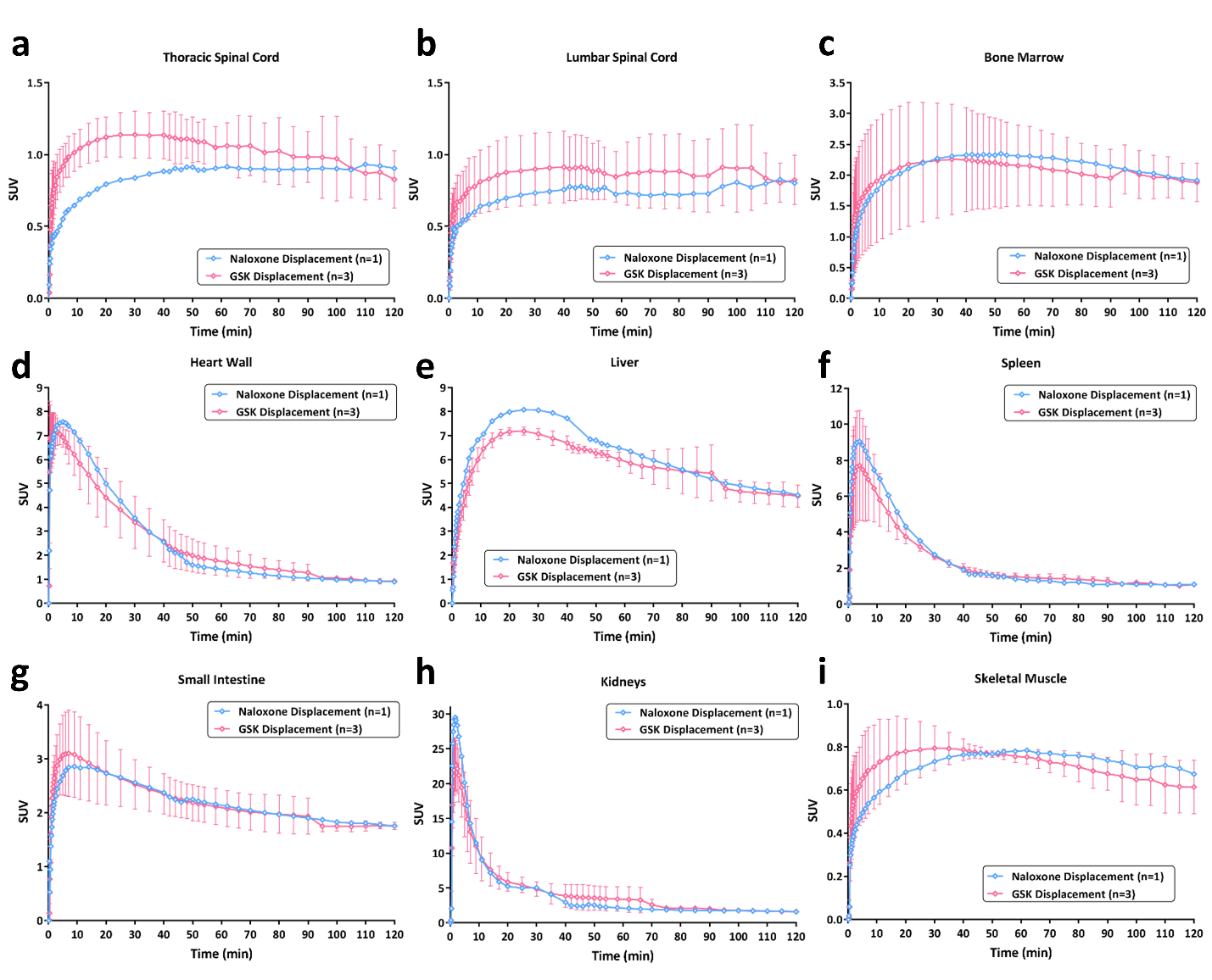


Supplementary Fig. 7. TACs of naloxone or GSK1521498 (GSK) displacement for spinal cord and peripheral organs. (**a**) thoracic spinal cord, (**b**) lumbar spinal cord, (**c**) spinal bone marrow, (**d**) heart wall, (**e**) liver, (**f**) spleen, (**g**) small intestine, (**h**) kidneys, and (**i**) skeletal muscle in baseline, retest, and naloxone pretreatment studies. Data points present as mean ± standard deviation.


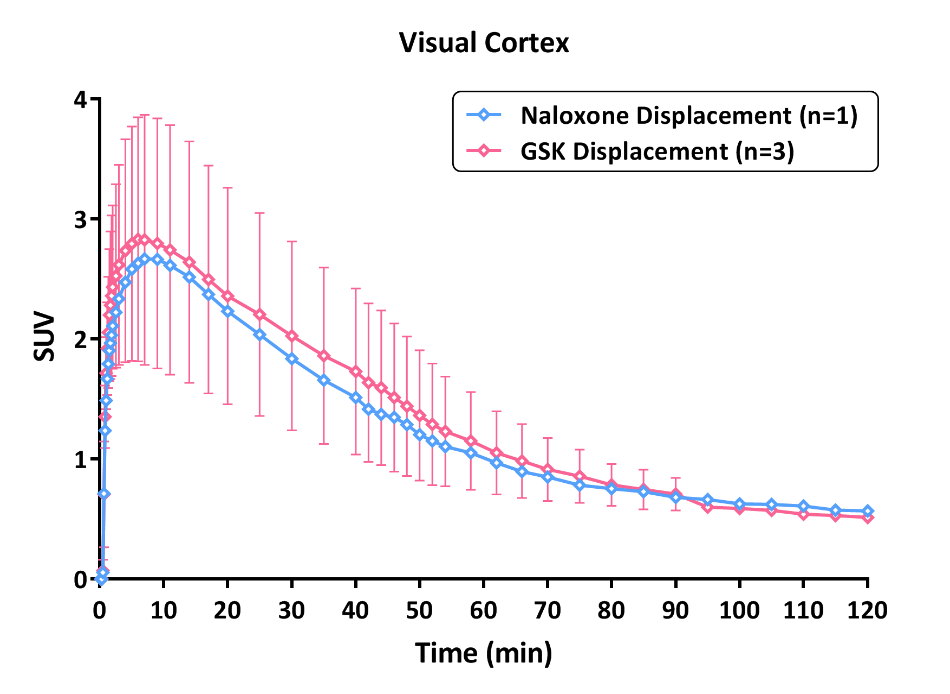


**Supplementary Fig. 8.** TACs of naloxone or GSK1521498 (GSK) displacement for visual cortex.


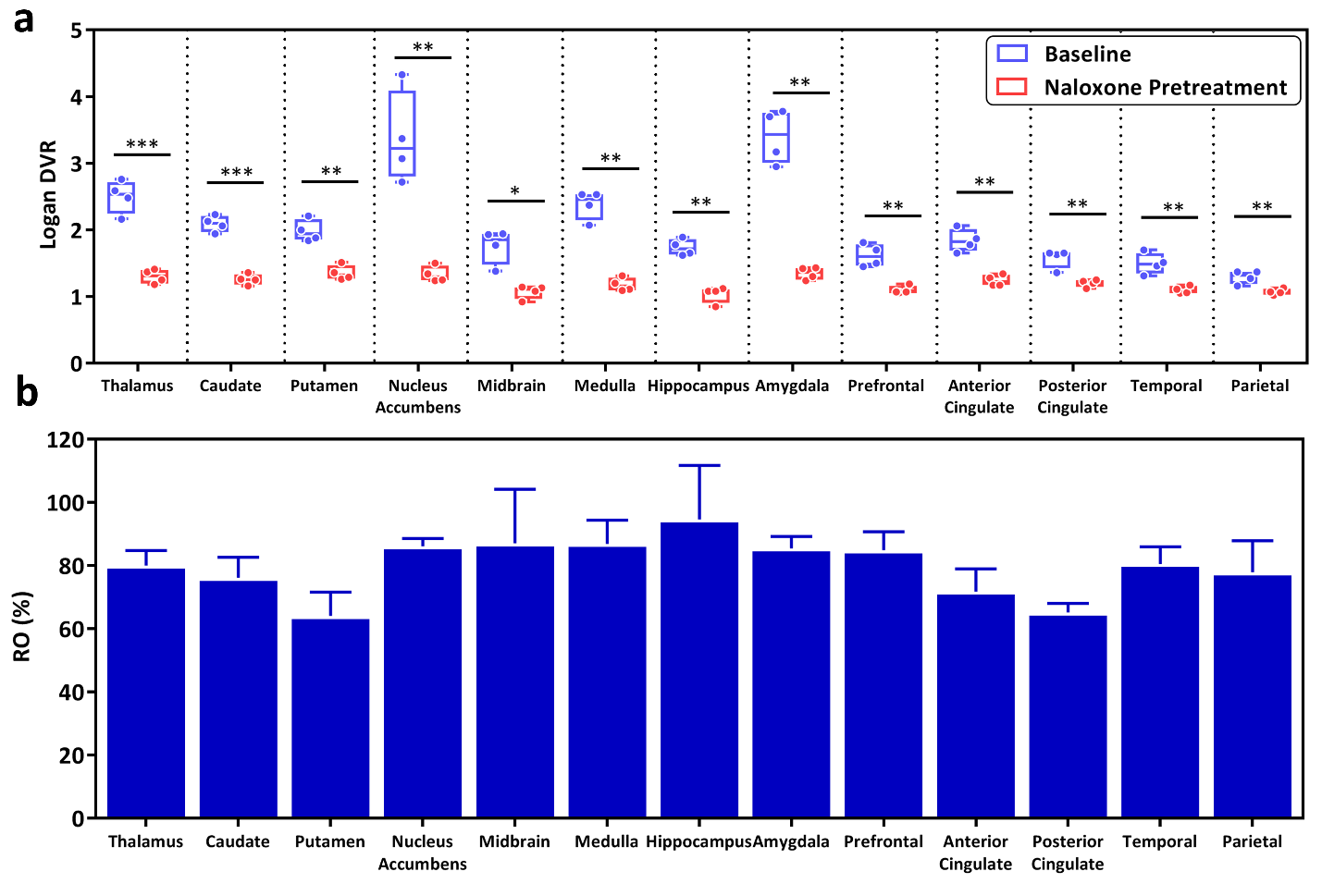


Supplementary Fig. 9. (**a**) Box plot of Logan DVR by using visual cortex/occipital as reference region for baseline and naloxone pretreatment. (**b**) Bar graph of %RO (mean ± standard deviation) in the different brain regions. Statistical significance of p value calculated by paired t-test: *** p < 0.001, ** p < 0.01, * p<0.05.

**Supplementary Table 4.** %RO of naloxone pretreatment and displacement studies for NHP-3

|  | Pretreatment | Displacement |
| --- | --- | --- |
| Thalamus | 87.9% | 81.8% |
| Caudate | 86.3% | 81.2% |
| Putamen | 77.8% | 71.5% |
| Nucleus accumbens | 92.0% | 90.7% |
| Midbrain | 91.7% | 84.5% |
| Medulla | 142.3% | 90.4% |
| Hippocampus | 93.5% | 87.3% |
| Amygdala | 91.3% | 91.5% |
| Prefrontal | 92.2% | 63.0% |
| Anterior cingulate | 85.6% | 82.7% |
| Posterior cingulate | 87.6% | 75.8% |
| Temporal | 93.2% | 65.2% |
| Parietal | 108.7% | 5.3% |


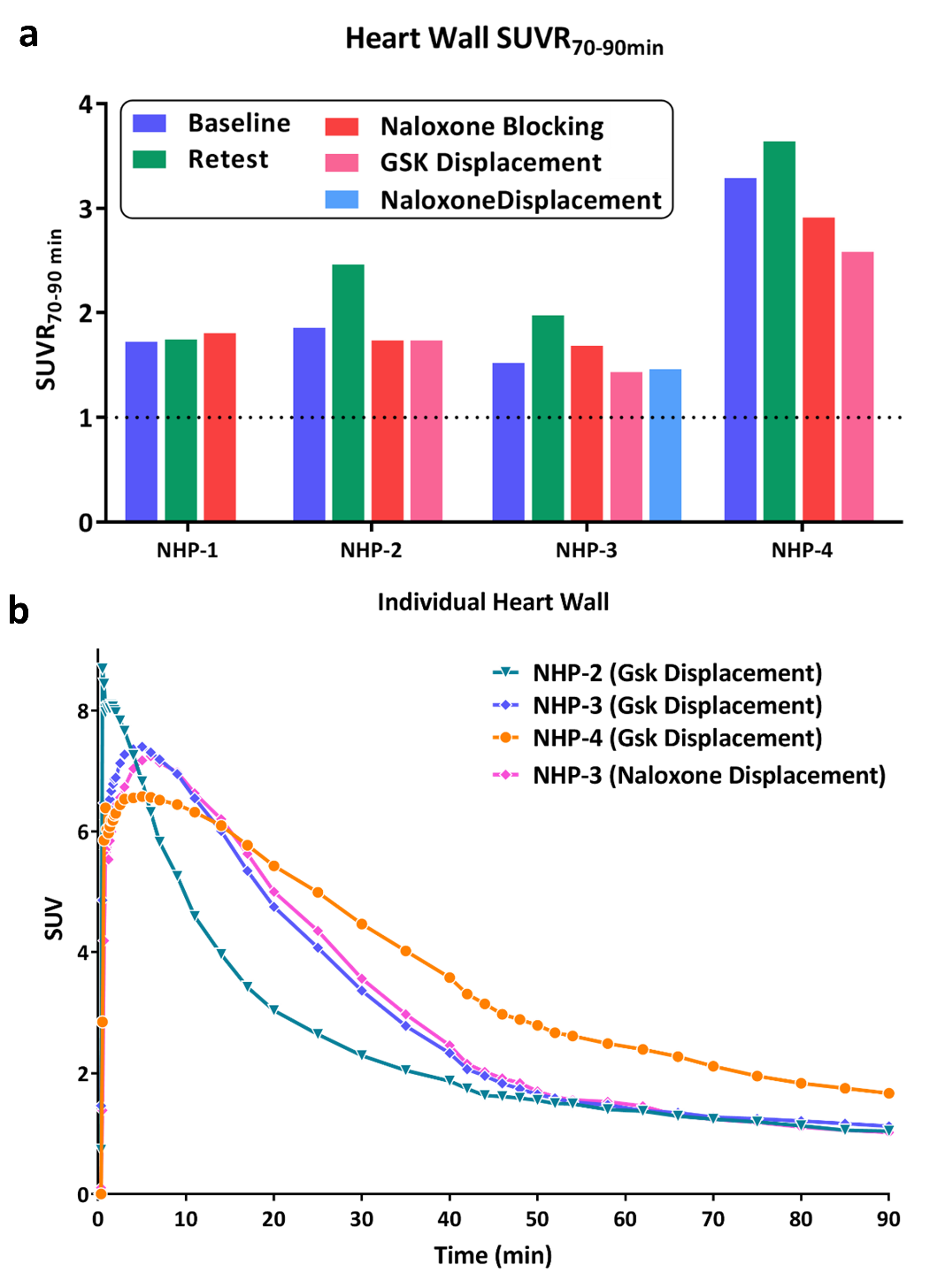


Supplementary Fig. 10. (**a**) Heart wall SUVR_70-90min_ of individual NHP in baseline, retest, naloxone pretreatment and displacement, and GSK1521498 displacement studies. (**b**) Individual heart wall TAC of naloxone or GSK1521498 displacement scan.
